# Combined PET/MRI: from Status Quo to Status Go. Summary Report of the Fifth International Workshop on PET/MR Imaging; February 15–19, 2016; Tübingen, Germany

**DOI:** 10.1007/s11307-016-0993-2

**Published:** 2016-08-17

**Authors:** D. L. Bailey, B. J. Pichler, B. Gückel, H. Barthel, A. J. Beer, R. Botnar, R. Gillies, V. Goh, M. Gotthardt, R. J. Hicks, R. Lanzenberger, C. la Fougere, M. Lentschig, S. G. Nekolla, T. Niederdraenk, K. Nikolaou, J. Nuyts, D. Olego, K. Åhlström Riklund, A. Signore, M. Schäfers, V. Sossi, M. Suminski, P. Veit-Haibach, L. Umutlu, M. Wissmeyer, T. Beyer

**Affiliations:** 1 Department of Nuclear Medicine, Royal North Shore Hospital, and Faculty of Health Sciences, University of Sydney, Sydney, Australia; 2 Werner Siemens Imaging Center, Department of Preclinical Imaging and Radiopharmacy, Eberhard-Karls-Universität, Tübingen, Germany; 3 Department of Interventional and Diagnostic Radiology, Eberhard-Karls-Universität, Tübingen, Germany; 4 Department of Nuclear Medicine, University Clinic, Leipzig, Germany; 5 Department of Nuclear Medicine, Ulm University, Ulm, Germany; 6 Division of Imaging Sciences and Biomedical Engineering, King’s College London, London, UK; 7 Moffitt Cancer Center, Tampa, USA; 8 Division of Imaging Sciences and Biomedical Engineering, Department of Cancer Imaging, King’s College London, London, UK; 9 Department of Nuclear Medicine, Radboud University, Nijmegen, The Netherlands; 10 Peter MacCallum Cancer Centre, Melbourne, Australia; 11 Department of Psychiatry and Psychotherapy, Medical University of Vienna, Vienna, Austria; 12 Division of Nuclear Medicine and clinical Molecular Imaging, Department of Radiology, University of Tübingen, Tübingen, Germany; 13 ZEMODI, Zentrum für Moderne Diagnostik, Bremen, Germany; 14 Department of Nuclear Medicine, Technical University Munich, Munich, Germany; 15 Strategy and Innovation Technology Center, Siemens Healthcare GmbH, Erlangen, Germany; 16 Department of Imaging and Pathology, Nuclear Medicine and Molecular Imaging, KU Leuven - University of Leuven, Leuven, Belgium; 17 Philips, 3000 Minuteman Road, Andover, MA 01810 USA; 18 Department of Diagnostic Radiology, Radiation Sciences, Umeå University/Norrlands University Hospital, Umeå, Sweden; 19 Nuclear Medicine Unit, Departments of Medical-Surgical Sciences and Translational Medicine, “Sapienza” University of Rome, Rome, Italy; 20 Department of Nuclear Medicine, University Hospital Münster and European Institute for Molecular Imaging, University of Münster, Münster, Germany; 21 Department of Physics and Astronomy, University of British Columbia, Vancouver, Canada; 22 GE Healthcare, PET/MR, Milwaukee, USA; 23 Department of Nuclear Medicine, University Hospital Zurich, Zurich, Switzerland; 24 Department of Diagnostic and Interventional Radiology and Neuroradiology, University Hospital Essen, Essen, Germany; 25 Department of Nuclear Medicine, University Hospital of Geneva, Geneva, Switzerland; 26 Center for Medical Physics and Biomedical Engineering, General Hospital Vienna, Medical University Vienna, 4L, Waehringer Guertel 18-20, 1090 Vienna, Austria

**Keywords:** PET/MRI, Combined imaging, Molecular imaging, PET/CT, PET, MRI, Quantification, Attenuation correction, Oncology, Neurology, Cardiology, Multi-parametric imaging

## Abstract

This article provides a collaborative perspective of the discussions and conclusions from the fifth international workshop of combined positron emission tomorgraphy (PET)/magnetic resonance imaging (MRI) that was held in Tübingen, Germany, from February 15 to 19, 2016. Specifically, we summarise the second part of the workshop made up of invited presentations from active researchers in the field of PET/MRI and associated fields augmented by round table discussions and dialogue boards with specific topics. This year, this included practical advice as to possible approaches to moving PET/MRI into clinical routine, the use of PET/MRI in brain receptor imaging, in assessing cardiovascular diseases, cancer, infection, and inflammatory diseases. To address perceived challenges still remaining to innovatively integrate PET and MRI system technologies, a dedicated round table session brought together key representatives from industry and academia who were engaged with either the conceptualisation or early adoption of hybrid PET/MRI systems. Discussions during the workshop highlighted that emerging unique applications of PET/MRI such as the ability to provide multi-parametric quantitative and visual information which will enable not only overall disease detection but also disease characterisation would eventually be regarded as compelling arguments for the adoption of PET/MR. However, as indicated by previous workshops, evidence in favour of this observation is only growing slowly, mainly due to the ongoing inability to pool data cohorts from independent trials as well as different systems and sites. The participants emphasised that moving from *status quo* to *status go* entails the need to adopt standardised imaging procedures and the readiness to act together prospectively across multiple PET/MRI sites and vendors.

## Introduction

This paper summarises discussions at the 2016 international positron emission tomography (PET)/magnetic resonance Imaging (MRI) workshop held in Tübingen, Germany. This is the fifth consecutive year that the faculty of the Eberhard-Karls-Universität and the Universitäts Klinikum Tübingen have jointly hosted this workshop, which is now firmly established as the most influential, vendor-independent international meeting to focus on the emerging applications and technological challenges of PET/MRI. This year, the workshop and meeting was attended by over 120 delegates from more than 20 countries, 50 % of whom came from outside Europe. Industry was also well represented with invited talks from the majority of the vendors involved in developing the hardware and software for PET/MRI.

One notable feature of the 2016 workshop was that there were more discussions on non-oncological applications of PET/MRI than in previous years. New areas of application that were addressed included inflammation and infection, cardiovascular applications, beta-cell function and mass in diabetes, neurotransmitters, radiomics, and tracking of radiolabelled cells. There are now over 100 PET/MRI systems worldwide either being operated or installed, a third of which were acquired in the last year. This growth rate should, of itself, broaden the range of applications which are investigated with PET/MRI.

The previous workshop suggested that PET/MRI with 2-deoxy-2-[^18^F]fluoro-D-glucose (FDG) should not try to compete with or replace [^18^F]FDG-PET/X-ray computed tomorgraphy (CT) in many oncological applications [1]. A role for [^18^F]FDG-PET/MRI was envisaged in paediatric oncology and for patients of reproductive potential to minimise exposure to ionising radiation from the CT component of a PET/CT study. Beyond this, however, it was thought that the most potentially productive areas for PET/MRI would be with non-[^18^F]FDG radiopharmaceuticals. A question was raised as to whether PET/MRI could be envisaged as the only PET system available in a facility, and most attendees agreed that it should not; the first choice, especially for oncological applications, would remain PET/CT mainly because of the shorter examination times and, secondly, because of easier interpretation by nuclear medicine physicians.

Participants at the meeting emphasised that a close collaboration between radiologists and nuclear medicine physicians helps the optimisation of acquisition protocols based on a deep knowledge of advanced MRI techniques. Naturally, patients with contraindications for undergoing an MRI scan may still require a PET/CT if a PET scan is indicated. The high cost of PET/MRI as compared to PET/CT was considered a major factor influencing the choice of imaging method, particularly in public and governmental institutions.

Each of the previous workshops has attempted to address the question as to what might be a key application for PET/MRI. This year, for the first time, prostate cancer imaging with [^68^Ga]PSMA has emerged as a tangible key application for PET/MRI. Of note, this was largely uncontested by those currently using PET/CT for this application.

As in previous summary reports, we attempt to provide succinct individual topic summaries and indicate major outcomes of the discussion boards. Likewise, we highlight progress achieved and comment on areas where only limited advances have been made. Finally, we will adhere again to the general conventions of previous reports to indicate progress (↑), steady state (↔) and regression (↓) in key aspects of PET/MRI. The key to the summary tables of changes in PET/MRI with respect to the status of the previous year is shown below.

**Table:** 

↑	Documented evidence of improvement in science and methodology
↗	Suggestion of improvement in methodology but requires further investigation
↔	No change but satisfactory status since previous workshop
↘	Little advancement in science and methodology despite previous recognition of need for improvement
↓	Less clear evidence than previously suggested

This year, the workshop commenced with discussion of two topics which are uppermost in the mind of many involved in the field, namely, “*How to move PET/MRI into clinical routine*” and “*From status quo to status go in PET/MRI*”.

## Discussion: How to Move PET/MRI into Clinical Routine

Since the introduction of combined, whole-body PET/MRI users have searched for the key clinical application [1–4]. This search has been conducted mainly by single institutions (rather than networks or by pooling data), and evidence in favour of, or against, identifying a specific indication as a key application has been weak. The topic of this opening session was chosen deliberately to tune in to recent scepticism regarding a broader role of combined PET/MRI in clinical routine [5, 6].

The speakers in the opening session were drawn both from private practice and from a university-based hospital, *i.e.* both with a strong interest in clinical PET/MRI (as opposed to research only). Some of the reasons given for the slow translation of PET/MRI into routine clinical practice included a general lack of guidelines and standardisation for the use of PET/MRI, ongoing lack of supporting evidence for potential clinical applications of PET/MRI, lack of protocol optimisation and advanced analysis software from collaborations between vendors and users, and unresolved issues relating to which professional groups should perform and report the scans. In an attempt to address some of these requirements, the *Japanese Radiologic Society*, the *Japanese Society of Nuclear Medicine*, and the *Japanese Society of Magnetic Resonance Imaging* have recently joined to produce a set of on-line guidelines for the use of PET/MRI [7].

At previous workshops there had been much discussion about the most appropriate MRI sequences to acquire in PET/MRI examinations [1, 4] and to try and reduce this number from what was *desirable* to what is *sufficient*. The protocols presented in this session for routine examinations have been optimised such that the total scan time has been minimised (Tables 1 and 2). In practice, it was reported that a five-bed position scan can be completed in 50–60 min while an extended nine-bed position scan (∼2 m axial extent) could take up to 90 min. Total running costs for operating a PET/MRI in Germany were estimated at approximately 100,000€ per calendar month. This presents a challenging business model in the absence of a satisfactory reimbursement scheme, as is currently the case in Germany.

**Table 1:** Example generic timeline for a PET/MRI oncological “whole-body” examination. Note that the PET acquisitions (6 min each) are approximately two to three times longer than for current PET/CT investigations, therefore, allowing for either a reduction in the amount of radiopharmaceutical administered or enhanced image quality using a standard amount administered (data courtesy of M.Lentschig, Bremen

Elapsed time [min)(	PET exam	MRI exam	MRI details			
0–2		Fast view scout				
3–4		Planning exam				
5–10	Pos1—pelvis	Pos1—pelvis	AC (DIXON) 19 s	T2w HASTE 42 s	DWI 120 s	T2w TRIM 150 s
11–12		Auto shim/breath hold				
13–18	Pos2—abdomen	Pos2—abdomen	AC (DIXON) 19 s	T2w HASTE 42 s	DWI 120 s	T2w TRIM 150 s
19–20		Auto shim/breath hold				
21–26	Pos3—thorax	Pos3—thorax	AC (DIXON) 19 s	T2w HASTE 42 s	DWI 120 s	T2w TRIM 150 s
27–28		Auto shim/breath hold				
29–34	Pos4—neck	Pos4—neck	AC (DIXON) 19 s	T2w HASTE 42 s	DWI 120 s	T2w TRIM 150 s
35–36		Auto shim/breath hold				
37–42	Pos5—head	Pos5—head	AC (DIXON) 19 s	T2w HASTE 42 s	DWI 120 s	T2w TRIM 150 s
43		Auto shim/breath hold				
44–47		Head-to-pelvis pre-contrast	T1 VIBE			
48–50		Liver	4× T1 VIBE			
51–54		Head-to-pelvis post-contrast	T1 VIBE

**Table 2:** Example timeline of a “fast protocol” for a whole-body PET/MRI for oncological indications. The total acquisition comprises four bed positions of 4-min emission time each and a post-contrast whole-body VIBE sequence (no PET), resulting in a total examination time on the order of that for a whole-body PET/CT scan (∼18 min) (data courtesy of L. Umutlu, Essen)

PET (min:s)	4:00	4:00	4:00	4:00	1:36
Anatomical region	Pelvis	Abdomen	Thorax	Head/neck	Whole body
MRI details	AC HASTE (axial) DWI	AC HASTE (axial) DWI	AC HASTE (axial) DWI	AC HASTE (axial) DWI	T1w VIBE + Gd-contrast

Other oncological applications that were suggested to have potential clinical application in the future using PET/MRI included breast cancer (where CT has virtually no role), prostate cancer (where multi-parametric MRI is extremely valuable [8–10]), colorectal carcinoma (where MRI was felt to have an advantage in detecting nodal involvement), melanoma (both for whole body and for brain), gynaecological cancers (specifically addressing the issue of loco-regional recurrence), and brain tumours (where CT contributes little); however, evidence to support these speculations does not currently exist.

A proposed variation on the existing protocol illustrated in Table 1 that was proposed was to begin the MRI acquisition during the uptake phase of the PET radiotracer, which is typically 60 min for [^18^F]FDG. While this would not reduce the total duration of imaging in the PET/MRI system, it would reduce the time that the subject was required to attend the clinic. An alternative protocol was proposed by a different experienced PET/MRI site to bring down overall image acquisition time to a length equivalent to that of a whole-body PET/CT (Table 2).

## Discussion: from Status Quo to Status Go in PET/MRI

While the previously reported session focussed on the potential clinical applications of PET/MRI (*i.e.* beyond research applications), this session focussed on what was required to move PET/MRI, into a role where the unique information that it enables can be integrated into routine practice and fully exploited. It was suggested that PET/CT could appropriately be described as “anatomically enhanced molecular imaging”. PET/MRI would need to provide more than this to justify its higher capital costs and reduced patient throughput compared to PET/CT. There was further discussion about the total length of the imaging protocol and the need for the vendors and users to work more closely in an effort to reduce this to the minimum possible, while still retaining the ability to better characterise tissue and pathologies than is possible with X-ray CT. Also echoing sentiments from the first session, it was felt that there was a need to identify where PET/MRI had an advantage over PET/CT studies (Table 3).

**Table 3:** List of PET/MRI and PET/CT preferred clinical indications as adopted at a long-standing PET/MRI site in Germany (adapted from Stephan Nekolla, Nuklearmedizinische Klinik der TU München, Germany)

Scan indication	PET/CT	PET/MRI
Neuroimaging		
Neurodegenerative disease or where MRI is standard of care	–	✓
Dynamic PET scans required	–	✓
MRI exclusion criteria met (valves, pacemakers, claustrophobia)	✓	–
Oncological Imaging		
Prostate primary staging, recurrence	✓	–
Prostate biopsy planning	–	✓
Prostate therapy	✓	–
Focus on liver	–	✓
Focus on lung	✓	–
Paediatric imaging	–	✓
MRI exclusion criteria met	✓	–
Problems lying supine for >30 min	✓	–
Cardiac imaging		
MRI exclusion criteria met	✓	–
All others	–	✓

Triaging patients to PET/CT or PET/MR, as it is done at one site by following the separation scheme laid out in Table 3, automatically leads to a discussion about whether PET/CT and PET/MRI should be considered as competing or complementary. Much of the initial work with PET/MRI was to establish whether it provided equivalent information to PET/CT in the more common indications. Many would argue that for the majority of the time PET/MRI probably does provide this. However, in most published work to date, the question of a complementary role was not one that has been given the same attention as the competitive role of the combined imaging modalities.

An example of how the complementary nature could be exploited would be for a PET/MRI examination to immediately follow a standard PET/CT procedure in selected patients, with the imaging objective being restricted to limited region of the body. The properties of the tissue encompassed by this volume would be more accurately characterised with multiple MRI sequences in combination with the radiotracer distribution obtained with PET/CT [11]. One scenario where this might be indicated could be intra-tumoural heterogeneity observed by PET with PET/CT imaging without the CT image being able to provide any further ability to characterise the tissue in question. A follow-on PET/MRI scan could focus on the lesion in question in a limited, regional PET/MRI investigation, which could be relatively rapid, but still provide useful information (*e.g.* diffusion coefficient, and oedema) about the tumour environment based on spatial characterisation from different sequences. However, the logistics of such an operation may be challenging.

Panellists also tried to put PET/MRI into the context of a wider spectrum of diagnostic techniques as related to future investigations in human disease. It was emphasised how the cost of genetic sequencing was decreasing rapidly, while the costs of many new therapies were increasing. A common theme was that “imaging needs to perform better than biopsy-based diagnosis”. The use of liquid biopsies (serum, urine) is increasing (*e.g.* transcriptomes and circulating DNA) and is likely to provide earlier information about disease status, recurrence, and progression. The unique role for imaging in this scenario will be to provide whole-body or regional information complementary to the biopsy or blood sample. To enhance this capability of the PET/MRI will require developments in multi-parametric imaging, advanced image analysis, and linking imaging to proteomics, metabolomics, and genomics. Each of these areas implies an expanded role for data mining and radiomics [12] to which PET/MRI is naturally suited. Finally, these advances need to be translated into clinical practice.

In summary, the opening sessions emphasised the following requirements for PET/MRI to move into mainstream clinical practice:

- Optimisation of PET/MRI protocols to achieve a balance between an abundance of image-based information and realising this in a practical imaging protocol (total examination time ≤45 min)
- Collaboration between the vendors and the users to achieve optimised acquisition protocols and software for analysis
- The development of specific guidelines for PET/MRI investigations
- Development of a suitable evidence base for the appropriate use of PET/MRI using appropriately designed trials acceptable to regulators and health technology agencies
- Exploitation of added clinical value combining multi-parametric PET/MRI data with multi-scale clinical, laboratory, histopathologic, and “–omics” data

## Dialogue Board 1: Neurosciences

### The Issues

Brain disorders are reported to cost more than $US1 trillion ($US1 × 10^12^) *p.a.* This is greater than the amount spent on cancer, cardiovascular disease, and diabetes combined [13]. PET occupies a unique role with its ability to non-invasively probe neurotransmitter systems with radiolabelled exogenous and endogenous molecules in a way that other modalities are unlikely to match in vivo. MRI provides new information about brain circuitry with the use of connectivity analysis for both structural and functional information. The combination of studying disrupted neurotransmitter pathways with PET and the downstream implications on connections within brain circuits therefore offers a potentially powerful paradigm. Examples include the interaction between dopamine release and executive function and the prediction of drug response using changes in extracellular serotonin on functional MRI (fMRI) and PET. Of particular interest will be studies that investigate the correlation between neurotransmitter release and brain networks that are activated in response to external stimuli. These studies require that the PET and MRI acquisitions be performed simultaneously. Such stimuli can be either pharmacological or, perhaps more importantly, habituation-dependent tasks with their novelty decreasing as the task is repeated or evoke a non-fully reproducible response (*e.g.* response to pain). The determination of such correlations is relevant to improve our understanding of diseases such as addiction, schizophrenia, or neurodegeneration.

### Recent Advances or Achievements

There has been a continuous, increasing focus on the role of PET/MRI in psychiatric disorders. In particular, the serotonergic system has been receiving greater attention given the fact that a number of highly selective and specific radioligands are available for the quantification of major receptors, transporters, and enzymes of this system. Examples of the uses for PET/MRI in this domain include (i) prediction of drug response using serotonergic radiotracers (drug occupancy, pharmacological interaction with fMRI, and hormonal interactions with brain function) and frequently prescribed antidepressants as selective serotonin reuptake inhibitors, (ii) the combination of PET + MRI + EEG to combine higher spatial and temporal resolution measurements, and (iii) the introduction of α_4_β_2_ cholinergic receptor radioligands [14]. Recent studies have also shown that dopamine agonists and antagonists induce opposite effects on cerebral blood flow providing important insights into neurovascular coupling [15, 16].

### New Evidence That Has Been Reported

A number of studies have now shown that the accuracy of PET image reconstruction from PET/MRI acquisitions using currently available methods for photon attenuation correction is acceptable. Previously, this was seen as a remaining barrier to more widespread introduction of PET/MRI brain studies. Today, the attenuation correction issue discussed in recent workshops [1] appears readily solved in the case of [^18^F]FDG brain imaging [17–19] and hence should be acceptable for neurotransmitter studies as the extended MRI sequences that can be used are compatible with dynamic, temporal data acquisition with PET in a single bed position. In addition, the morphological imaging from MRI can be used to define intra-cerebral arteries where the profile of the arterial blood input signal can be determined for use in kinetic models of PET radioligands, if suitable corrections for tracer metabolites, when needed, can be devised.

New evidence on PET image reconstruction using time-of-flight (ToF) information to further improve the accuracy of attenuation correction appears to be promising [20–23]. In parallel to the neuroreceptor PET imaging procedure, information on neurodegeneration can be obtained from concomitant arterial spin labelling MRI [24] and voxel-based morphometry of anatomical MRI data [25]. Overall progress indicators for PET/MRI in neurology are given in Table 4.

**Table 4:** Progress indicators for PET/MRI in neurology

	2012	2013	2014	2015	2016
Improved understanding of brain physiology and function through the use of combined PET/MRI	↔	↗	↗	↗	↗
Methodological progress for improved quantification of PET/MRI neurological examinations (AC, IDIF, SUV)	↔	↔	↗	↗	↗
MR-based motion correction for routine clinical use	↓	↘	↔	↔	↔

### Future Challenges

A major technical hurdle during complex neuroimaging protocols is the limited ability to integrate multiple datasets into appropriate kinetic and neural models. An appropriate framework is needed for the quantitative combination of parameters from different modalities, and it is hoped that field of *radiomics* can offer additional insights and support.

One advantageous feature of integrated PET/MRI is the possibility of on-line movement correction of the acquired PET data by MRI-based movement detection [26]. In the field of neuroreceptor studies, this technological gain should be tested for its potential improvement of dopaminergic imaging in movement disorders, like Parkinson’s disease. Future neuroreceptor PET/MRI research activities should also involve studying the opioid system and its effect on brain haemodynamics, *e.g.* in pain disorders [27], as well as proof-of-concept studies on neurotransmitter drug testing.

One challenge for simultaneous dynamic imaging arises from the different temporal resolution of PET and fMRI and the fact that kinetic modelling using dynamic PET data usually assumes a steady-state situation, whereas MRI can be used to study evoked responses over a short time frame (*e.g.* BOLD technique). However, recent studies demonstrated promising new applications of simultaneous measurements of fMRI and fPET with a timing resolution of minutes in both methods, thus comparing tasks that have been classically restricted to fMRI or ASL [28–30]. Thus, the comparison of functional and molecular “connectomes” of the human brain now seems to be within reach with PET/MRI.

Finally, more advanced PET data analysis methods such as for neurotransmitter PET will need to be further developed to fully exploit multi-parameter information provided by PET/MRI [31, 32].

## Dialogue Board 2: Cardiovascular Diseases

### The Issues

The previous year’s workshop proposed that cardiovascular PET/MRI studies would develop a significant clinical role by combining pre-existing examinations already carried out on separate PET and MRI systems into one convenient examination [1]. However, at this workshop, the presenters focussed more on identifying new areas of application for PET/MRI where studies on either stand-alone PET or MRI were not likely to answer the clinical question, and hence PET/MRI would provide unique information. In this regard, the move from the research domain to the clinic has not progressed as anticipated, with new areas of application (as discussed below) still being investigated. Areas that were discussed included imaging inflammation in both the vessel wall (plaque imaging) and the myocardium, and imaging of cardiac innervation. For vascular imaging, MRI is currently accepted as the gold standard for the whole-body characterisation of the arterial vessel wall in determining the presence, size, and type of atherosclerotic plaques. However, the addition of PET should help to further identify the vulnerable plaque [33].

For myocardial tissue characterisation, quantitative T1 mapping was also discussed as a surrogate marker of therapeutic response and outcome in diffuse myocardial processes such as post-infarction fibrosis or in cardiomyopathies or storage diseases [34, 35]. PET measurements may supplement these in identifying areas of acute inflammation and identifying metabolic changes using [^18^F]FDG.

### Recent Advances or Achievements

Although there is a clear vision for a unique application of PET/MRI in cardiovascular imaging [36], recent advances have been few. This can be explained in part by the technical challenges of imaging small and moving structures, such as the arterial vessel wall and the myocardium. To address some of these challenges, current research has been focused on MRI-based cardiac and respiratory motion compensation to improve both MRI and PET image qualities as well as PET-based quantification [37]. The following list of tracers were considered to be of potential interest for clinical cardiac PET imaging in combination with MRI:

- [^18^F]NaF for imaging vulnerable plaque
- [^64^Cu]DOTA-Octreotate for imaging macrophages
- [^68^Ga]Pentixafor for imaging CXCR4 expression in inflammation
- [^18^F]BR351 for imaging of matrix metalloprotease activity in inflammation
- [^18^F]FDG-labelled leucocytes for imaging inflammation
- [^18^F]LMI1195 for imaging cardiac innervation

### New Evidence That Has Been Reported

PET/MRI has been mainly used in combination with [^18^F]FDG to identify and characterise vascular inflammation in the carotid artery [38, 39]. In general, these studies focussed on protocol development, the feasibility of the integrated PET/MRI approach, and quantification issues. First studies have been described to integrate PET imaging of innervation with structural and functional MRI imaging for the characterisation of post-infarct myocardium [40] (Table 5). PET/MRI imaging of (vulnerable) plaque in the coronary arteries, even though clinically desirable, still suffers from limited robustness of MRI for this specific application, thus limiting a broader clinical implementation of dedicated PET/MRI protocols for coronary inflammation and atherosclerosis [41].

**Table 5:** Progress indicators for PET/MRI in cardiovascular diseases (CVD)

	2012	2013	2014	2015	2016
Resolution of methodological issues for CVD imaging (MR-AC, motion correction)	NA	↗	↗	↔	↔
Develop analysis tools for standard CVD applications	NA	↔	↔	↔	↔
Identification of key parameters/biomarkers from PET and MRI to avoid redundancy in PET/MRI data	NA	↔	↗	↗	↗
Standardised imaging protocols	NA	↔	↔	↔	↘

Combined [^18^F]FDG PET/MRI early after myocardial infarction following successful intervention revealed a high predictive value for the assessment of inflammatory tissue state during adverse remodelling of the left ventricle [42]. In addition, novel tracers targeting these processes more specifically are becoming available [43]. These strategies have the potential to optimise patient-specific medical interventions and also to prospectively assess novel therapies using quantitative measures.

### Future Challenges

The panellists suggested that some of the remaining technical challenges for PET/MRI included the reduction of truncation artefacts, the incorporation of respiratory and cardiac motion compensation, the shortening of the lengthy acquisitions, the development of radiopharmaceuticals that maximise the plaque-to-blood contrast in the vascular system, and the quantification of MRI signals.

In spite of the prediction of the previous year’s workshop regarding the clinical role of PET/MRI in cardiovascular imaging, attendees conceded that at this stage cardiovascular PET/MRI was still a research tool. This reflection was partially based on the fact that most of the processes in cardiovascular disease (atherosclerosis, myocardial infarction and cardiomyopathies) can be described only to a limited extent through animal models.

## Dialogue Board 3: Oncology

### The Issues

Oncological imaging with [^18^F]FDG has been the focus of PET/MRI since its inception, largely based on the success of PET/CT in this area. The view that has emerged from the Tübingen workshops over the preceding years is that the future of PET/MRI is in areas of oncology where PET/CT currently has little or no role. Examples include cancers of the breast, prostate, pancreas, and some indications for imaging the liver. It was proposed that combined multi-modality imaging could provide three general types of data:

(i) Complementary information
(ii) Confirmatory information
(iii) Redundant information

In this context, PET/MRI could be considered to be a single modality (neither PET-only nor MR-only), thereby assuming more and more the role of a "problem solving" tool. Reviewing five years of PET/MRI in oncology, panellists concluded that PET/MRI performed equally well as PET/CT in many areas [6] but at the cost of increasing infrastructure needs and longer acquisition times. In paediatric imaging, in particular, no clear diagnostic superiority of PET/MRI over PET/CT has been demonstrated to date, however, the studies can be performed with a significantly reduced radiation exposure.

### Recent Advances or Achievements

One area where it appears that PET/MRI has now demonstrated superior diagnostic performance to PET/CT in oncology is in prostate cancer [^68^Ga]-PSMA imaging [44]. There was a notable increased degree of discussion at this workshop on the role for loco-regional PET/MRI, potentially using previous imaging to direct the PET/MRI to a particular region of the body for a more thorough characterisation. It was noted by one of the panellists that, if this approach (*i.e.* regional PET/MRI) were implemented, it could be achieved with a significantly reduced radiation exposure from the PET study by reducing the amount of radiotracer administered, as the MRI sequences would be the rate-limiting step while the PET was acquired simultaneously over a longer period than usual. Specifically, in standard TOF-PET/MRI, clinical [^18^F]FDG doses can be lowered by 40–50 % compared to standard TOF-PET/CT while maintaining equivalent image quality [45]. One potential example for single station imaging with PET/MRI and ultra-low radiotracer doses administered would be breast imaging. Here, effective patient exposure from the combined examination of around 1 mSv appears achievable. With such indications, PET/MRI could be opened up for a wider field of clinical oncological indications (Table 6).

**Table 6:** Progress indicators for PET/MRI in oncology, including paediatric imaging

	2012	2013	2014	2015	2016
Definition of key clinical applications	↔	↔	↗	↗	↗
Diagnostic quality of PET in PET/MRI equivalent to PET quality in PET/CT	↔	↔	↗	↗	↗
Resolving quantitative bias from MR-AC	↘	↔	↔	↔	↗
Clinical data available on diagnostic accuracy of PET(/MRI) in oncology	↔	↔	↗	↔	↗
PET/MRI protocol standardisation	↓	↔	↘	↔	↔
Clinical evidence on the usefulness of PET/MRI in paediatric oncology	↔	↗	↗	↗	↔
Reduced radiation exposure as a key driver for PET/MRI of children	↗	↑	↑	↔	↘
Initial results of a complementary role of advanced MRI techniques for restaging of lymphoma patients	↔	↔	↗	↔	↔

### New Evidence That Has Been Reported

Since the last meeting, there have been further published studies of the diagnostic potential of PET/MRI in cancers of the cervix [46], endometrium [47], colorectal [48], breast [49], head and neck [50], and thyroid and metastatic prostate disease [51] in comparison to PET/CT and for response prediction in gastric cancer [52]. However, the number of patients studied remains relatively small.

### Future Challenges

The challenge remains to define areas of clinical application where combined PET/MRI will provide new and unique information. Prostate cancer imaging or the reduction of radiation dose alone was thought unlikely to justify the cost of PET/MRI for most users. An important challenge to be addressed was the need to simplify MRI acquisition protocols to avoid sequences that provide no incremental value to that already provided by PET, thus, focussing rather on selecting sequences that provide superior information than that provided by the CT component of PET/CT.

## Dialogue Board 4: Infection and Inflammation

### The Issues

This was a new topic introduced for the first time at this workshop. Nuclear medicine imaging has had a long history of infection imaging using agents such as [^67^Ga]citrate, radiolabelled white blood cells (WBCs), [^111^In]Oxine, bacterial imaging agents, and [^18^F]FDG. In sterile inflammatory processes, a large number of potential targets exist that may be amenable to imaging such as GLUT, TSPO, SSTR, COX, MMP, CB_2_R, FPR, CD20, CD25 (IL-2R), TNFR, VCAM-1, VAP-1, and α_2_β_3_ [53]. The proposed advantages of PET/MRI for imaging infection and inflammation include the ability to image the whole body, and to use the PET, in particular, to identify the most active areas for further MRI characterisation. Alternatively, a whole-body PET/CT study can be considered the method of choice for searching for an infective focus or disseminated foci followed by a loco-regional PET/MRI imaging procedure of the respective areas of interest, as promoted in a similar fashion in the Dialogue Board on oncology.

Some of the potential clinical areas of application named for combined PET/MRI included soft tissue inflammation, GI inflammation (inflammatory bowel disease) [54, 55], cardiovascular processes such as acute myocarditis and sarcoidosis, rheumatoid arthritis [56], type-1-diabetes [57], giant cell arteritis, and spondylodiscitis [58]. It was noted that, yet again, CXCR4 and CD25 are expressed on most infiltrating cells in many of these processes and hence provide a PET target.

### Recent Advances or Achievements

This is an emerging area and hence clinical data to support this application area still being investigated (Table 7).

**Table 7:** Progress indicators for PET/MRI in infection and inflammation imaging

	2012	2013	2014	2015	2016
Improved tissue characterisation by combined PET/MRI	–	–	–	–	↗
Development of new radiopharmaceuticals for PET use in general	–	–	–	–	↗
Standardised imaging protocols	–	–	–	–	↔
Standardised image interpretation criteria	–	–	–	–	↔
Definition of key clinical applications	–	–	–	–	↗

### New Evidence That Has Been Reported

Recent insight into the role of PET/MRI in inflammation has been published [53]. Some centres already use [^18^F]FDG-PET/CT for inflammation/infection imaging although in most cases standardised image interpretation criteria are absent [58].

### Future Challenges

The panellists concurred that cell labelling and tracking using PET would be extremely helpful in these application areas. This implies the use of radionuclides with longer half-lives than the main ones currently used (F-18, C-11, Ga-68). Candidates that potentially fulfil this requirement include Cu-64, Zr-89 and I-124 providing these will not modify cell viability or function. Alternatively, new peptides and/or cytokines labelled with conventional PET short half-life radionuclides might allow us to target cells in different clinical situations [59–61]. This suggestion may foreshadow the need for an increase in future radiopharmaceutical production.

## Dialogue Board 5: Emerging Areas

### The Issues

The second new session was dedicated to emerging areas that may have synergy with PET/MRI. In the introduction to the session the audience was reminded that PET/MRI users must be “*more than just photographers*”, implying that PET/MRI should provide a deeper characterisation of the physiological processes involved and not just be used to identify sites of disease (*e.g.* “hot spot localisation”). The two areas chosen for focus in this session were the emerging field of radiomics and the role of PET/MRI in pancreatic β-cell imaging and diabetes.

### Recent Advances or Achievements

Radiomics refers to “the extraction and analysis of large amounts of advanced quantitative imaging features with high throughput from medical images obtained with computed tomography, positron emission tomography or magnetic resonance imaging” [62]. It is based on the proposition that “images are more than pictures; they are data” [12]. PET/MRI provides an extremely useful platform for radiomics development based on multi-modality imaging from a large number of complementary signals. PET/CT, in contrast, would be restricted to essentially two components only, the PET signal and the CT morphology, whereas MRI is able to provide a multiplicity of sequences that can probe different aspects of the tissue micro-environment. An example of a potential new target was carbonic anhydrase 9 (CA-IX) and its increased expression at the advancing edge of cancerous tissues [63, 64].

Secondly, in this session, the discussion turned to studying the pancreas with PET/MRI. The emerging burden of diabetes in developed societies is often referred to as an epidemic. PET/MRI should be an ideal tool to study a metabolic disorder such as diabetes. In recent years, evidence has been growing that beta cell loss is not the sole and leading pathophysiological process in the pancreas that finally leads to insufficient production of insulin. Instead, loss of the capacity of the beta cells to produce and release insulin appears to play a more important role, caused by or preceding de-/trans-differentiation and apoptosis. Measuring and following beta cell mass in relation to beta cell function (*i.e*. insulin production, storage capacity, and release) during the course of the disease and in response to treatments would therefore be a major asset in diabetes research [65, 66]. Data were presented to indicate that PET/MRI can measure both β-cell mass and β-cell function, using manganese contrast MRI for function (namely insulin release) and [^68^Ga]Exendin to assess the mass [65, 67].

### New Evidence That Has Been Reported

Both areas are at the cutting edge of developments and may benefit from PET/MRI studies in the future (Table 8). While radiomics has mostly been applied to cancer to date this may be extended into other areas with PET/MRI, and the field of diabetes and metabolism is essentially a new focus for imaging with PET/MRI. Both methods rely on quantitative imaging approaches.

**Table 8:** Multi-parametric imaging and emerging areas

	2012	2013	2014	2015	2016
Fully integrated PET/MRI exclusively offers the largest variety of multi-parametric biomarkers	↔	↗	↑	↑	↑
Validation of advanced multi-parametric biomarkers in clinical research (beyond “image fusion”)	↘	↔	↗	↗	↔
Contributions of small animal imaging to the understanding of multi-parametric biomarkers	↔	↗	↑	↑	↗
Using standardised approaches for assessing the accuracy of PET/MRI and towards multi-parametric image analysis	–	–	–	↗	↔

### Future Challenges

Data management will be a challenge when combining the large number of signals produced by PET/MRI over millions of voxels in the case of whole-body studies. Robust statistical techniques will be required to extract essential information and protect against type I errors. In addition, individuals with skills that are able to span the biological to the statistical and imaging fields will need to be recruited, trained, and retained.

It is becoming increasingly apparent that minor gains in diagnostic accuracy, or even significant improvements in this regard, will be insufficient alone to ensure reimbursement of new imaging techniques, particularly if these add significantly to the cost of diagnosis. Adding an expensive imaging modality, like PET/MRI, to existing imaging paradigms is likely to face significant hurdles purely on economic grounds. Therefore, the design of novel diagnostic approaches, which provide superior diagnosis at comparable cost or that improve allocation of expensive therapies, is required. Most trials of new imaging modalities have focussed on demonstrating the incremental diagnostic information after conventional imaging tests have been performed. If the cost of the technique is high and the diagnostic gains are small, cost effectiveness is unlikely.

**Fig. 1:**
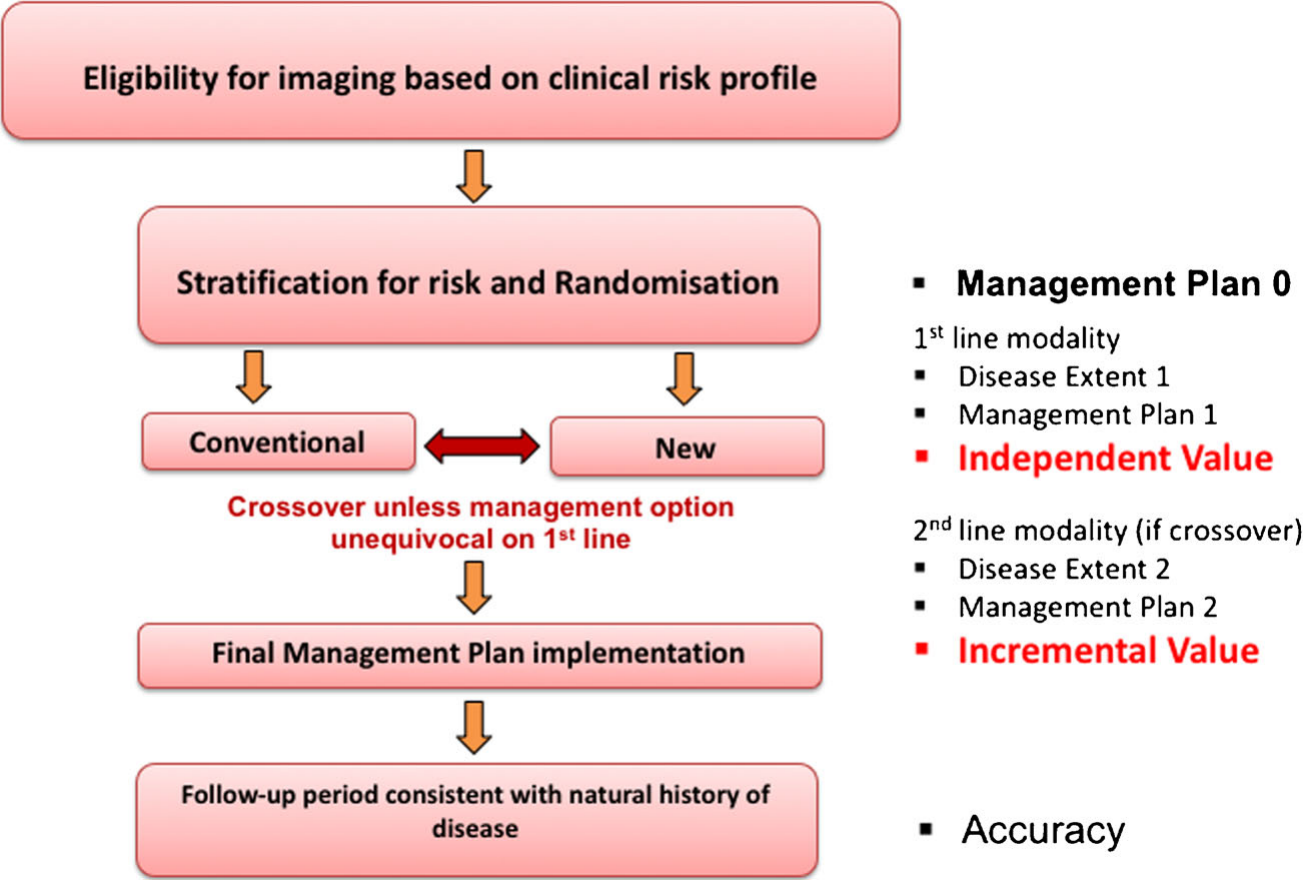
Flow diagram of proposed randomised control trial (RCT) for evaluating novel imaging paradigms. Rather than demonstrating incremental value from single-injection, dual-imaging PET/CT-PET/MRI comparisons, an added value needs to be assessed from a management plan implementation (courtesy of R Hicks, Peter Mac Cancer Centre Melbourne, Australia).

An alternative approach is to address the independent diagnostic value of doing the more accurate but more expensive test instead of conventional imaging and offsetting any differential cost by savings in avoidance of additional investigations or inappropriate therapeutic interventions. A randomised crossover design has been developed by researchers at the Peter MacCallum Cancer Centre in Australia to compare conventional and experimental imaging paradigms for independent and incremental utility (Fig. 1). By randomly assigning patients to either conventional or experimental imaging, the independent diagnostic utility is defined by those cases in which the definitive management plan is decided upon by the initial test performed. For example, if the primary test indicates systemic metastasis and excludes all loco-regional therapies and mandates systemic therapy, the patient would not crossover. Alternatively, unless there is a definitive exclusion of alternative therapies on the basis of this scan, the patient would crossover to the alternative paradigm. Incremental diagnostic information is defined by a change in final management plan on the basis of the second study. If the management decision remains unchanged, the study is defined as having no impact. By contrast, if the second investigation changes treatment intent or modality (high impact), or delivery of a previously planned therapeutic modality without change in treatment intent (medium impact), an incremental value is demonstrated.

It should be noted that almost all imaging studies look at the incremental value of new tests by applying them in a subgroup of patients based on eligibility criteria defined by conventional imaging. This methodology was first utilised to assess the incremental utility of PET imaging in patients with lung cancer who had no definite evidence of metastatic disease on conventional imaging [68, 69] and was subsequently adopted by the National Oncological PET Registry (NOPR) [70], which led to wider reimbursement of PET and PET/CT. In the proposed study design, the appropriateness of the final decision is validated by a composite measure of accuracy determined from the combination of pathological validation, serial imaging, and most importantly, by the final outcome. The aggregate costs of arriving at a final management plan for each strategy, combined with patient preference and final clinical outcomes of patients, are then used to measure cost effectiveness and cost utility.

In addition to clinical impact, a further dimension of imaging that needs to be considered is the ability of a test to characterise disease. This goes beyond simply finding disease and defining a treatment course. Tests that can better stratify outcome within a group of patients with the same type and extent of disease receiving the same therapy can be said to provide added value. Imaging as a predictive or prognostic biomarker is an area of application where PET/MRI may transcend either modality alone, while use of highly specific PET tracers and multi-parametric MRI may also surpass [^18^F]FDG-PET/CT. Related discussions can be found in [71].

## Roundtable Discussion—How to Build and Sustain Innovation

In this session, Industry met with academia and clinical users to address the issue of how PET/MRI can achieve its full potential, beyond where it is today, by utilising the myriad of tools and biological targets it already has available. The beginning point for any innovation needs to be good science and the ability to see the larger picture. The industry participants reiterated that invention is not the same as innovation and that innovation is something that people want to adopt “and are prepared to pay for”. In the context of healthcare, innovation was seen as “turning good ideas into something that can be used in the clinic” or as one of the panellists put it “*innovation* is an *invention* that creates added *value*”. Thus, innovation would ideally produce better outcomes and increased access for patients while helping to contain or decrease costs.

The key to a sustainable use of PET/MRI was seen to be the development of expanded data analysis methods that would provide new, multi-parametric information about disease, rather than just providing more images to be visually inspected. From the vendors’ perspective the three main challenges in healthcare today are a demographic shift towards an aging population, an underserved demand for state-of-the-art healthcare (particularly in emerging countries), and rising costs in existing healthcare systems. In light of these challenges, PET/MRI today appears more of a challenge than a solution.

As the discussion ensued, the question which arose was whether PET/MRI had been developed to solve a particular problem or whether it had been simply assumed that the combination of these two high-end medical imaging modalities would synergistically produce an innovative device which users would be prepared to pay for. Naturally, the answer to that question varied with the background of the responder. Going by the definition of innovation, panellists and attendees agreed on the potential of PET/MRI but that, as it stands, PET/MRI cannot be called an innovative technique quite yet, given the limited proof of added value [6].

## Summary

The topics of the Tübingen PET/MRI workshops have evolved over the years in an attempt to identify and discuss the most salient contemporary questions (Table 9). This fifth workshop has shown that in oncology a clear demarcation between [^18^F]FDG-PET/CT and newer studies with non-[^18^F]FDG PET/MRI has emerged. Prostate cancer imaging may well prove to be the first key application in oncology for PET/MRI, but it remains to be seen whether the incremental value is confined to the pelvis or can be applied efficiently to whole-body protocols. In cardiovascular disease, despite the promise and current use of both PET and MRI in cardiovascular investigation, PET/MRI is still seen mainly as a research tool but may be well suited to the dedicated imaging of atherosclerotic plaque and myocardial inflammatory processes in the future. Neuroscience studies with PET/MRI had begun to emerge where the contemporaneous measurement of the PET and MRI signals are seen as essential, for example, in joint PET and MRI functional activation studies, drug challenges, and functional connectivity investigations but these remain, like cardiology, largely in the domain of research. The studies are virtually impossible to reproduce on separate imaging systems during separate investigations.

**Table 9:** Progress indicators for key applications for PET/MRI

	2012	2013	2014	2015	2016
Paediatric oncology is a key application of PET/MRI	↗	↗	↑	↑	↘
Dementia is a key application of PET/MRI	↗	↑	↑	↑	↗
Neuro-Oncology is a key application of PET/MRI	↗	↗	↗	↔	↔
Cardiovascular imaging is a key application of PET/MRI	↔	↔	↔	↗	↔
Multi-centre evaluation of clinical PET/MRI	↓	↘	↘	↘	↘
Multi-parametric imaging is a key driver for PET/MRI	↔	↗	↗	↗	↑

The new areas that were discussed for the first time at the meeting this year included infection and inflammation imaging, functional imaging of the pancreas in diabetes, and the emerging science of radiomics. Many of these areas will require new approaches to data analysis and will provide challenges for the future.

**Fig. 2:**
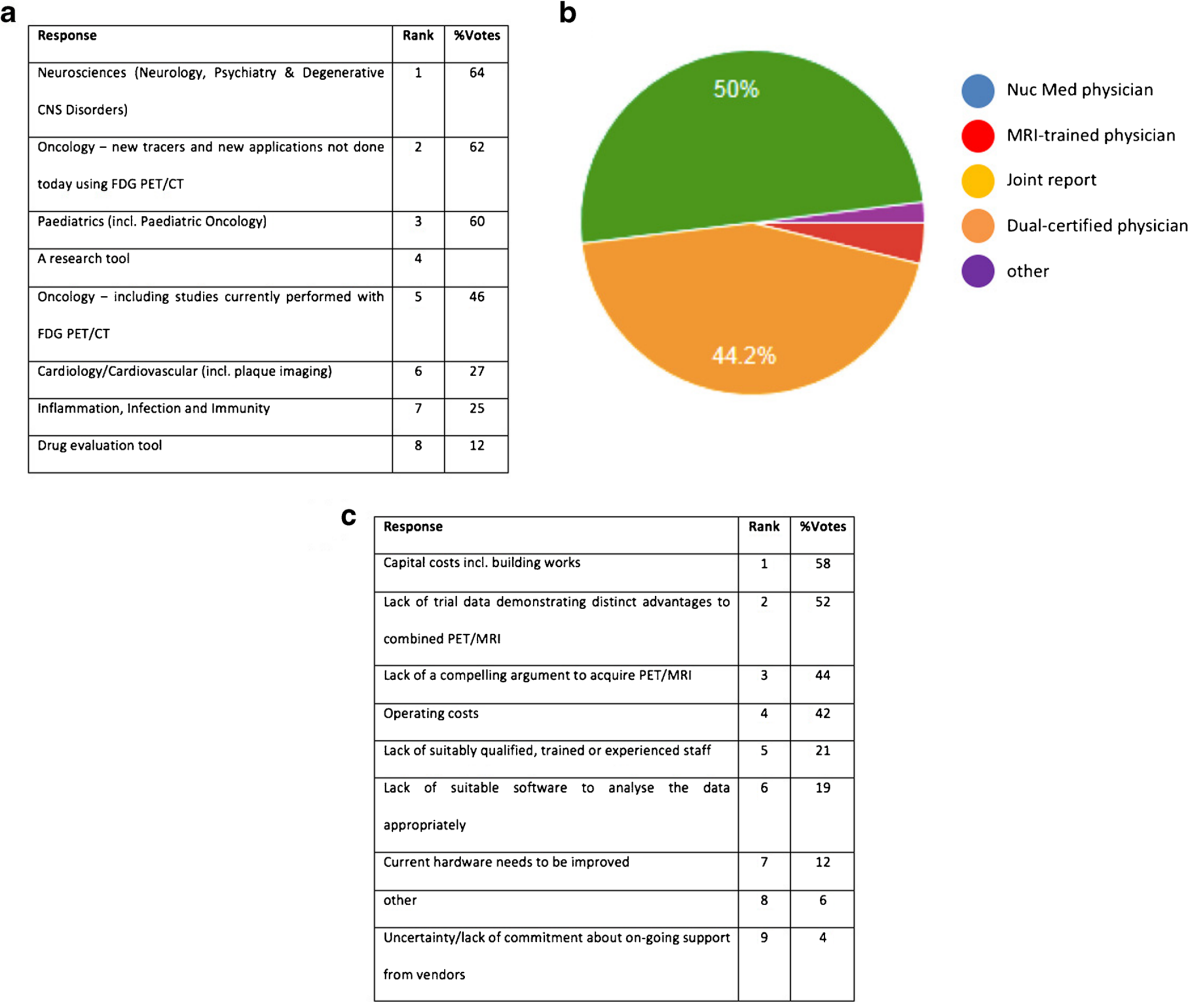
Poll results from fifth PET/MRI workshop participants: **a** What do you think will be the main application for PET/MRI (select up to three)? **b** Who should read PET/MRI examinations in clinical practice? and **c** What are the greatest barrier to bringing PET/MRI to the clinic (select up to three)? (questions courtesy of Dr Sarah Bond, Mirada Medical, UK).

During one of the sessions the audience was posed a series of questions related to commonly addressed aspects of PET/MRI implementation. Subsequent to the meeting, the questions were formalised into a survey and participants were invited to respond (52 (43 %) did respond) to the questions that were asked (Fig. 2) and show clearly that the field is moving to a more sophisticated view on the use of PET/MRI, largely for new and unique imaging applications. It is clear that developments in closely related fields—from radiochemistry to radiomics—will continue to allow PET/MRI to explore new horizons well into the future to impact on our understanding of not only the nature of human disease but also the nature of the disease in the individual human.

## References

[1] Bailey DL, Pichler BJ, Guckel B (2015). Combined PET/MRI: multi-modality multi-parametric imaging is here: summary report of the 4th international workshop on PET/MR imaging; February 23-27, 2015, Tubingen, Germany. Mol Imaging Biol.

[2] Bailey DL, Barthel H, Beyer T (2013). Summary report of the first international workshop on PET/MR imaging, March 19-23, 2012, Tubingen, Germany. Mol Imaging Biol.

[3] Bailey DL, Barthel H, Beuthin-Baumann B (2014). Combined PET/MR: where are we now? Summary Report of the Second International Workshop on PET/MR Imaging April 8-12, 2013, Tubingen, Germany. Mol Imaging Biol.

[4] Bailey DL, Antoch G, Bartenstein P et al (2015) Combined PET/MR: the real work has just started. Summary Report of the Third International Workshop on PET/MR Imaging; February 17-21, 2014, Tubingen, Germany. Mol Imaging Biol 17(3):297–312 10.1007/s11307-014-0818-0 PMC4422837 25672749

[5] Czernin J, Ta L, Herrmann K (2014). Does PET/MR imaging improve cancer assessments? Literature evidence from more than 900 patients. J Nucl Med.

[6] Spick C, Herrmann K, Czernin J (2016). ^18^F-FDG PET/CT and PET/MRI perform equally well in cancer: evidence from studies on more than 2,300 patients. J Nucl Med.

[7] Tateishi U, Nakamoto Y, Murakami K, et al. Guidelines for the clinical use of 18F-FDG-PET/MRI 2012 (Ver 1.0). http://www.jsnm.org/files/pdf/guideline/2013/PET-MRI_Guideline_Ver1.0_Part1_Tateishi_U.pdf.

[8] Johnson LM, Choyke PL, Figg WD, Turkbey B (2014). The role of MRI in prostate cancer active surveillance. BioMed Res Intl.

[9] Johnson LM, Turkbey B, Figg WD, Choyke PL (2014). Multiparametric MRI in prostate cancer management. Nature Reviews Clin Oncol.

[10] Johnson LM, Rothwax JT, Turkbey B (2014). Multiparametric magnetic resonance imaging of the prostate aids detect lesion progression. J Comput Assist Tomogr.

[11] Hicks RJ, Lau EW (2009). PET/MRI: a different spin from under the rim. Eur J Nucl Med Mol Imaging.

[12] Gillies RJ, Kinahan PE, Hricak H (2016). Radiomics: images are more than pictures, they are data. Radiology.

[13] Smith K (2011). Trillion-dollar brain drain. Nature.

[14] Sabri O, Becker GA, Meyer PM (2015). First-in-human PET quantification study of cerebral alpha4beta2* nicotinic acetylcholine receptors using the novel specific radioligand (-)-[(18)F]Flubatine. Neuroimage.

[15] Sander CY, Hooker JM, Catana C (2013). Neurovascular coupling to D2/D3 dopamine receptor occupancy using simultaneous PET/functional MRI. Proc Natl Acad Sci U S A.

[16] Sander CY, Hooker JM, Catana C, Rosen BR, Mandeville JB (2016). Imaging agonist-induced D2/D3 receptor desensitization and internalization in vivo with PET/fMRI. Neuropsychopharmacol.

[17] Ladefoged C, Benoit D, Law I (2015). PET/MR attenuation correction in brain imaging using a continuous bone signal derived from UTE. Eur J Nucl Med Mol Imaging Physics.

[18] Burgos N, Cardoso MJ, Thielemans K (2014). Attenuation correction synthesis for hybrid PET-MR scanners: application to brain studies. IEEE Trans Med Imaging.

[19] Burgos N, Cardoso MJ, Thielemans K (2014). Attenuation correction synthesis for hybrid PET-MR scanners: validation for brain study applications. EJNMMI physics.

[20] Defrise M, Rezaei A, Nuyts J (2012). Time-of-flight PET data determine the attenuation sinogram up to a constant. Phys Med Biol.

[21] Defrise M, Rezaei A, Nuyts J (2014). Transmission-less attenuation correction in time-of-flight PET: analysis of a discrete iterative algorithm. Phys Med Biol.

[22] Rezaei A, Defrise M, Bal G (2012). Simultaneous reconstruction of activity and attenuation in time-of-flight PET. IEEE T Med Imaging.

[23] Rezaei A, Defrise M, Nuyts J (2014). ML-reconstruction for TOF-PET with simultaneous estimation of the attenuation factors. IEEE T Med Imaging.

[24] Zhang K, Herzog H, Mauler J (2014). Comparison of cerebral blood flow acquired by simultaneous [15O]water positron emission tomography and arterial spin labeling magnetic resonance imaging. J Cereb Blood Flow Metab.

[25] Choi H, Cheon GJ, Kim HJ (2016). Gray matter correlates of dopaminergic degeneration in Parkinson’s disease: a hybrid PET/MR study using (18) F-FP-CIT. Hum Brain Mapp.

[26] Catana C, Drzezga A, Heiss WD, Rosen BR (2012). PET/MRI for neurologic applications. J Nucl Med.

[27] Wey HY, Catana C, Hooker JM (2014). Simultaneous fMRI-PET of the opioidergic pain system in human brain. Neuroimage.

[28] Riedl V, Bienkowska K, Strobel C (2014). Local activity determines functional connectivity in the resting human brain: a simultaneous FDG-PET/fMRI study. J Neurosci.

[29] Hahn A, Gryglewski G, Nics L, et al. (2016) Quantification of task-specific glucose metabolism with constant infusion of [18F]FDG. J Nucl Med 57:(In Press). 10.2967/jnumed.116.176156 27390156

[30] Villien M, Wey HY, Mandeville JB (2014). Dynamic functional imaging of brain glucose utilization using fPET-FDG. Neuroimage.

[31] Normandin MD, Schiffer WK, Morris ED (2012). A linear model for estimation of neurotransmitter response profiles from dynamic PET data. Neuroimage.

[32] Normandin MD, Koeppe RA, Morris ED (2012). Selection of weighting factors for quantification of PET radioligand binding using simplified reference tissue models with noisy input functions. Phys Med Biol.

[33] Wildgruber M, Swirski FK, Zernecke A (2013). Molecular imaging of inflammation in atherosclerosis. Theranostics.

[34] Moon JC, Messroghli DR, Kellman P (2013). Myocardial T1 mapping and extracellular volume quantification: a Society for Cardiovascular Magnetic Resonance (SCMR) and CMR Working Group of the European Society of Cardiology consensus statement. J Cardiovasc Magn Reson.

[35] Kellman P, Hansen MS (2014). T1-mapping in the heart: accuracy and precision. J Cardiovasc Magn Reson.

[36] LaForest R, Woodard PK, Gropler RJ (2016). Cardiovascular PET/MRI: challenges and opportunities. Cardiol Clinics.

[37] Grimm R, Furst S, Souvatzoglou M (2015). Self-gated MRI motion modeling for respiratory motion compensation in integrated PET/MRI. Medical Image Anal.

[38] Skeoch S, Williams H, Cristinacce P (2015). Evaluation of carotid plaque inflammation in patients with active rheumatoid arthritis using (18)F-fluorodeoxyglucose PET-CT and MRI: a pilot study. Lancet.

[39] Li X, Heber D, Rausch I et al (2016) Quantitative assessment of atherosclerotic plaques on F-FDG PET/MRI: comparison with a PET/CT hybrid system. Eur J Nucl Med Mol Imaging 43:1503–1512 10.1007/s00259-016-3308-6 PMC4906060 26816195

[40] de Haan S, Rijnierse MT, Harms HJ (2015). Myocardial denervation coincides with scar heterogeneity in ischemic cardiomyopathy: a PET and CMR study. J Nucl Cardiol DOI.

[41] Dweck MR, Puntman V, Vesey AT, Fayad ZA, Nagel E (2016). MR imaging of coronary arteries and plaques. JACC Cardiovasc Imaging.

[42] Rischpler C, Dirschinger RJ, Nekolla SG (2016). Prospective evaluation of 18F-fluorodeoxyglucose uptake in postischemic myocardium by simultaneous positron emission tomography/magnetic resonance imaging as a prognostic marker of functional outcome. Circ Cardiovasc Imaging.

[43] Rischpler C, Nekolla SG, Kossmann H (2016). Upregulated myocardial CXCR4-expression after myocardial infarction assessed by simultaneous GA-68 pentixafor PET/MRI. J Nucl Cardiol.

[44] Eiber M, Weirich G, Holzapfel K (2016). Simultaneous Ga-PSMA HBED-CC PET/MRI improves the localization of primary prostate cancer. Eur Urol.

[45] Queiroz MA, Delso G, Wollenweber S (2015). Dose optimization in TOF-PET/MR compared to TOF-PET/CT. PLoS One.

[46] Binse I, Poeppel TD, Ruhlmann M (2016). Imaging with (124)I in differentiated thyroid carcinoma: is PET/MRI superior to PET/CT?. Eur J Nucl Med Mol Imaging.

[47] Vrachimis A, Burg MC, Wenning C (2016). [18F]FDG PET/CT outperforms [18F]FDG PET/MRI in differentiated thyroid cancer. Eur J Nucl Med Mol Imaging.

[48] Sawicki LM, Grueneisen J, Schaarschmidt BM (2016). Evaluation of (18)F-FDG PET/MRI, (18)F-FDG PET/CT, MRI, and CT in whole-body staging of recurrent breast cancer. Eur J Radiol.

[49] Grueneisen J, Nagarajah J, Buchbender C (2015). Positron emission tomography/magnetic resonance imaging for local tumor staging in patients with primary breast cancer: a comparison with positron emission tomography/computed tomography and magnetic resonance imaging. Invest Radiol.

[50] Schaarschmidt BM, Heusch P, Buchbender C (2016). Locoregional tumour evaluation of squamous cell carcinoma in the head and neck area: a comparison between MRI, PET/CT and integrated PET/MRI. Eur J Nucl Med Mol Imaging.

[51] Freitag MT, Radtke JP, Hadaschik BA (2016). Comparison of hybrid 68Ga-PSMA PET/MRI and 68Ga-PSMA PET/CT in the evaluation of lymph node and bone metastases of prostate cancer. Eur J Nucl Med Mol Imaging.

[52] Lee DH, Kim SH, Im SA, Oh DY, Kim TY, Han JK (2015) Multiparametric fully-integrated 18-FDG PET/MRI of advanced gastric cancer for prediction of chemotherapy response: a preliminary study. Eur Radiol 26:2771–2778 10.1007/s00330-015-4105-5 26615555

[53] Wu C, Li F, Niu G, Chen X (2013). PET imaging of inflammation biomarkers. Theranostics.

[54] Catalano OA, Gee MS, Nicolai E (2016). Evaluation of quantitative PET/MR enterography biomarkers for discrimination of inflammatory strictures from fibrotic strictures in Crohn disease. Radiology.

[55] Pellino G, Nicolai E, Catalano OA (2016). PET/MR versus PET/CT imaging: impact on the clinical management of small-bowel Crohn’s disease. J Crohns Colitis.

[56] Chaudhari AJ, Bowen SL, Burkett GW (2010). High-resolution (18)F-FDG PET with MRI for monitoring response to treatment in rheumatoid arthritis. Eur J Nucl Med Mol Imaging.

[57] Signore A, Capriotti G, Chianelli M (2015). Detection of insulitis by pancreatic scintigraphy with 99mTc-labeled IL-2 and MRI in patients with LADA (Action LADA 10). Diabetes Care.

[58] Glaudemans AW, Quintero AM, Signore A (2012). PET/MRI in infectious and inflammatory diseases: will it be a useful improvement?. Eur J Nucl Med Mol Imaging.

[59] Di Gialleonardo V, Signore A, Glaudemans AW, Dierckx RA, De Vries EF (2012). N-(4-18F-fluorobenzoyl)interleukin-2 for PET of human-activated T lymphocytes. J Nucl Med.

[60] Malmberg C, Ripa RS, Johnbeck CB (2015). 64Cu-DOTATATE for noninvasive assessment of atherosclerosis in large arteries and its correlation with risk factors: head-to-head comparison with 68Ga-DOTATOC in 60 patients. J Nucl Med.

[61] Vag T, Gerngross C, Herhaus P (2016). First experience with chemokine receptor CXCR4-targeted PET imaging of patients with solid cancers. J Nucl Med.

[62] Kumar V, Gu Y, Basu S (2012). Radiomics: the process and the challenges. Magn Reson Imaging.

[63] Lloyd MC, Cunningham JJ, Bui MM, Gillies RJ, Brown JS, Gatenby RA (2016) Darwinian dynamics of intratumoral heterogeneity: not solely random mutations but also variable environmental selection forces. Cancer Res 76:3136–3144 10.1158/0008-5472.CAN-15-2962 PMC5384728 27009166

[64] Tafreshi NK, Lloyd MC, Proemsey JB (2016). Evaluation of CAIX and CAXII expression in breast cancer at varied O2 levels: CAIX is the superior surrogate imaging biomarker of tumor hypoxia. Mol Imaging Biol.

[65] Gotthardt M, Eizirik DL, Cnop M, Brom M (2014). Beta cell imaging—a key tool in optimized diabetes prevention and treatment. Trends Endocrinol Metab.

[66] Eriksson O, Laughlin M, Brom M et al (2016) In vivo imaging of beta cells with radiotracers: state of the art, prospects and recommendations for development and use. Diabetologia 59:1340–1349 10.1007/s00125-016-3959-7 27094935

[67] Mikkola K, Yim CB, Fagerholm V (2014). 64Cu- and 68Ga-labelled [Nle(14), Lys(40)(Ahx-NODAGA)NH2]-exendin-4 for pancreatic beta cell imaging in rats. Mol Imaging Biol.

[68] Kalff V, Hicks RJ, MacManus MP (2001). Clinical impact of (18)F fluorodeoxyglucose positron emission tomography in patients with non-small-cell lung cancer: a prospective study. J Clin Oncol.

[69] Hicks RJ, Kalff V, MacManus MP (2001). The utility of (18)F-FDG PET for suspected recurrent non-small cell lung cancer after potentially curative therapy: impact on management and prognostic stratification. J Nucl Med.

[70] Hillner BE, Liu D, Coleman RE (2007). The National Oncologic PET Registry (NOPR): design and analysis plan. J Nucl Med.

[71] Hofman MS, Hicks RJ (2015). Moving beyond “lumpology”: PET/CT imaging of pheochromocytoma and paraganglioma. Clin Cancer Res.

